# Preclinical Characterization of Recombinant Human Tissue Kallikrein-1 as a Novel Treatment for Type 2 Diabetes Mellitus

**DOI:** 10.1371/journal.pone.0103981

**Published:** 2014-08-06

**Authors:** Tadeusz Kolodka, Matthew L. Charles, Arvind Raghavan, Ilian A. Radichev, Christina Amatya, Jacob Ellefson, Alexei Y. Savinov, Abhijeet Nag, Mark S. Williams, Mark S. Robbins

**Affiliations:** 1 DiaMedica USA, Inc., Minneapolis, Minnesota, United States of America; 2 Sanford Project/Children’s Health Research Center, Sanford Research, Sioux Falls, South Dakota, United States of America; 3 Invitek, Inc., Hayward, California, United States of America; Bristol Heart Institute, University of Bristol, United Kingdom

## Abstract

Modulation of the kallikrein-kinin system (KKS) has been shown to have beneficial effects on glucose homeostasis and several other physiological responses relevant to the progression of type 2 diabetes mellitus (T2D). The importance of bradykinin and its receptors in mediating these responses is well documented, but the role of tissue kallikrein-1, the protease that generates bradykinin *in* *situ*, is much less understood. We developed and tested DM199, recombinant human tissue kallikrein-1 protein (rhKLK-1), as a potential novel therapeutic for T2D. Hyperinsulinemic-euglycemic clamp studies suggest that DM199 increases whole body glucose disposal in non-diabetic rats. Single-dose administration of DM199 in obese *db/db* mice and ZDF rats, showed an acute, dose-dependent improvement in whole-body glucose utilization. Sub-acute dosing for a week in ZDF rats improved glucose utilization, with a concomitant rise in fasting insulin levels and HOMA1-%B scores. After cessation of sub-acute dosing, fasting blood glucose levels were significantly lower in ZDF rats during a drug wash-out period. Our studies show for the first time that DM199 administration results in acute anti-hyperglycemic effects in several preclinical models, and demonstrate the potential for further development of DM199 as a novel therapeutic for T2D.

## Introduction

According to the World Health Organization (2012) there are more than 340 million people affected by diabetes worldwide, of which 90% suffer from type 2 diabetes mellitus (T2D). Although new classes of therapeutics such as glucagon-like 1 peptide receptor (GLP-1) agonists, dipeptidyl peptidase-4 (DPP-4) inhibitors and sodium-glucose co-transport (SGLT2) inhibitors have been recently approved, there is still a need for therapies with novel mechanisms of action that can reduce hyperglycemia and ameliorate the complications of diabetes [1].

The kallikrein-kinin system (KKS) includes the serine protease tissue kallikrein-1 (KLK-1), its natural biological substrates, kininogens, and the peptide cleavage products, bradykinin (BK) and lys-bradykinin. The KKS is best characterized by its role in mediating inflammation, the regulation of blood pressure and cardiovascular function (reviewed in [2]–[5]). However, in the context of T2D pathogenesis and progression, several reports suggest a role for the KKS in insulin sensitization and glucose homeostasis. BK acting through the bradykinin 2 receptor (BKR2) has been shown to increase insulin-induced glucose uptake, stimulate insulin-induced translocation of glucose transporter 4 (GLUT4), and to potentiate insulin-induced phosphorylation of the insulin receptor and insulin receptor substrate-1 [6]–[8]. Glucose uptake and insulin sensitivity in normal rats are dramatically reduced by administration of BKR2 antagonists [9], while insulin resistance and impaired glucose tolerance are more pronounced in kininogen-deficient rats compared to wild-type controls [10].

Human tissue kallikrein-1, a ubiquitous 238 amino acid glycoprotein, exists as a heterogeneous mixture of glycoforms due to variable glycosylation at three potential sites. In gene therapy experiments, ectopic KLK-1 expression in fructose-induced pre-diabetic hypertensive rats, significantly reduced hypertension and hyperinsulinemia [11]. In streptozotocin-induced diabetic rats, adenoviral expression of KLK-1 significantly reduced blood glucose, plasma triglyceride and cholesterol levels [12]. In the same rat model, recombinant adeno-associated viral delivery of KLK-1 reversed insulin resistance [13].

While these studies suggest various anti-diabetic benefits of KLK-1 gene delivery, the expressed KLK-1 protein was not characterized in terms of dose, glycoform profile or activity. Additionally, gene-therapy is not at present a viable approach as an anti-diabetic therapeutic modality. Herein we report evidence that administration of purified recombinant human KLK-1 (DM199) elicits improvements in fasting blood glucose levels, and improves whole-body glucose disposal in preclinical animal models of T2D. The results suggest that DM199 has potential for further development as a novel T2D therapeutic.

## Materials and Methods

### Preparation and characterization of DM199

DM199, recombinant human tissue kallikrein-1 (rhKLK-1), was produced from Chinese hamster ovary (CHO) cells expressing a gene encoding the full length pre-pro-protein for human tissue kallikrein-1 (NP_002248.1). Following harvest and clarification, the supernatant containing secreted pro-KLK-1 was treated with recombinant trypsin (Roche Diagnostics, Germany) to generate active KLK-1 The active KLK-1 was further purified under aseptic conditions by column chromatography and filtration essentially as described [14], [15]. N-terminal Edman sequencing of purified DM199 confirmed that the protein was exclusively active KLK-1, free of the pro-KLK-1 heptapeptide. The specific activity of DM199 was measured *in* *vitro* by cleavage of the substrate D-Val-Leu-Arg-7 amido-4-trifluoromethylcoumarin (D-VLR-AFC, FW 597.6; Sigma, Cat #V2888 or Ana Spec Inc Cat #24137). When D-VLR-AFC was hydrolyzed, the free AFC produced in the reaction was quantified by fluorometric detection (excitation 360 nm, emission 460 nm). DM199 activity was determined by comparing the relative activity of a DM199 sample to the porcine kininogenase standard acquired from the National Institute for Biological Standards and Control (NIBSC Product No. 78/543). For this standard, the assigned potency is 22.5 international units (IU) per 20 µg ampoule of porcine pancreatic kininogenase.

### Animal Research and Ethics Statement

All animal studies were carried out in strict accordance with the recommendations in the Guide for the Care and Use of Laboratory Animals of the National Institutes of Health. Protocols were approved by the Sanford Animal Care and Use Committee at Sanford Research Institute (Permit #16-03-12) and the Animal Care and Use Committee at Invitek, Inc. (Permit #A4629-01). All surgeries were performed under anesthesia as described below, and all efforts were made to minimize suffering. At the end of each experiment, animals were euthanized by CO_2_ asphyxiation.

### Hyperinsulinemic-Euglycemic Clamp

Male Sprague Dawley rats (8–9 weeks old; 250–275 g) were obtained from Harlan Laboratories (Indianapolis, IN) and acclimated for seven days under standard husbandry conditions (food and water *ad libitum*). Rats were randomized and anesthetized with an intraperitoneal injection of ketamine-xylazine cocktail. The right jugular vein and left carotid artery were catheterized externally through an incision in the skin flap. The catheterized animals were allowed to recover for five days. After five days of recovery, animals were fasted for 12 hours and injected subcutaneously (s.c.) with DM199 or phosphate buffered saline (PBS). After 30 minutes, a hyperinsulinemic-euglycemic clamp (HEC) was started (t = 0 min) with continuous infusion of human insulin (Humulin, Eli Lilly, Indianapolis, IN) at a constant rate of 4 mU/kg/min. At the same time a 20% glucose (Sigma-Aldrich) solution was infused at a variable rate (glucose infusion rate), with adjustments every 10 minutes to maintain a target blood glucose level of 115±5 mg/dl. Both insulin and glucose were infused through the catheterized right jugular vein and blood glucose levels were monitored from the catheterized carotid artery. Arterial blood glucose levels and plasma insulin levels were measured from 50 µl samples prior to infusion at t = −120, −90, −30, −15 and 0 minutes and then at every 10 minutes for 120 minutes (t = 120), using a Glucose meter (Accu-Chek Roche Diagnostics, Indianapolis, IN) and a rat insulin ELISA kit. At t = 120 min, the experiment was terminated and all animals euthanized by CO_2_ asphyxiation.

### Fasting Blood Glucose and Oral Glucose Tolerance Tests in Female *db/db* Mice

Ten-week old female *db/db* mice (B6.BKS(D)-*Lepr^db^*/J) were purchased from The Jackson Laboratories (Bar Harbor, ME) and acclimated for 2 weeks under standard husbandry conditions (food and water *ad libitum*). The diabetic status of the mice was verified by non-fasting blood glucose >250 mg/dl for three consecutive days prior to stratification. All blood glucose measurements were performed on a 5 µl sample of blood obtained by tail-vein prick, without replacement. An Ascensia Elite 1 one-touch blood glucose monitor (Bayer, Mishawaka, IN) was used for measurement. To determine the effects of the test compounds on fasting blood glucose (FBG) levels, animals were randomly divided into experimental groups and after an overnight fast were subjected to a single s.c. injection of vehicle (PBS), insulin glargine (Lantus), or DM199. Blood glucose levels were measured prior to drug administration and then at 30 minute intervals for the next 4 hours.

After 7 days of recovery, the same cohorts of *db/db* mice underwent an oral glucose tolerance test (OGTT). Animals were fasted overnight (12 hours) and test compounds were administered thirty minutes prior to gavage with 2 g/kg of glucose (Sigma-Aldrich) dissolved in distilled deionized water. Blood glucose levels were measured as described above at −30, 0, 30, 60, 90, 120, 150, 180, 210, and 240 minutes after glucose challenge, and the experiment was terminated.

### Fasting Blood Glucose and Oral Glucose Tolerance Tests in Male ZDF *fa/fa* Rats

Male ZDF *fa/fa* rats (ZDF-*Lepr^fa^*/CRL) 10 weeks of age were obtained from Charles River (Wilmington, MA), and housed individually under standard husbandry conditions (food and water *ad libitum*). After nine days of acclimatization, rats were randomized into 4 dosing groups according to their FBG levels. Thereafter FBG was monitored daily at approximately the same time each morning for 14 days. On days 1, 4 and 7, rats received an s.c. injection of DM199 or PBS following the morning FBG measurement. Two hours after treatment (t = 0 min), rats were gavaged with 2 g/kg glucose (Sigma-Aldrich) in sterile water. Blood glucose levels were measured at 0, 30, 60, 90, and 120 minutes after treatment, and the experiment was terminated at t = 120 min. For each insulin and glucagon measurement, approximately 100 µl blood samples were drawn through a cut tail vein, without replacement. Blood samples were collected into EDTA containing microtainer tubes (BD Biosciences) and 600 KIU of aprotinin (Sigma-Aldrich) per mL of blood was immediately added. Plasma was prepared by centrifugation and stored at −80°C until analysis by ELISA for insulin (Crystal Chem, Inc. Downers Grove, IL) and glucagon (R & D Systems, Inc. Minneapolis, MN) following manufacturer’s instructions. For all blood glucose measurements, a 5 µl sample of blood obtained by tail-vein prick, without replacement, was spotted on a test strip and read using an Accu-Chek Aviva Plus glucose meter (Roche Diagnostics, Indianapolis, IN). The HOMA1-%B measure of beta cell function was calculated by the formula, ([Fasting plasma insulin µU/ml] * 360)/([Fasting blood glucose mg/dl]−63). The HOMA1-IR estimate of insulin resistance was calculated by the formula, [Fasting plasma insulin (µU/ml) * Fasting blood glucose (mg/dl)/405] [16].

### Data Analyses

To test for statistical significance, a one-way ANOVA of the means followed by either a Tukey test or a Student’s t-test with Bonferroni post-hoc correction was carried out. Differences between groups were considered significant at *p<*0.05. All data are presented as the mean +/− SEM.

## Results

### Hyperinsulinemic Euglycemic Clamp (HEC) in Sprague Dawley Rats

**Figure 1:**
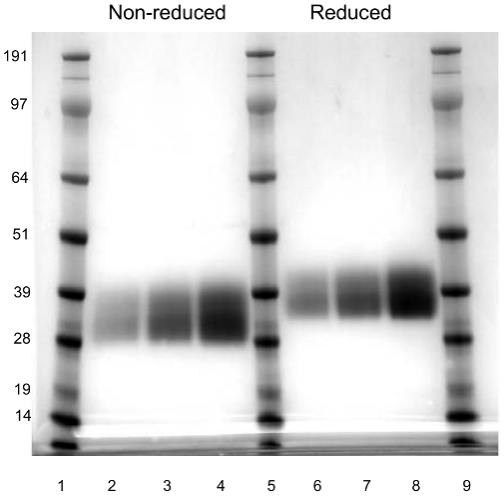
Purified DM199. 1, 2 and 4 µg of purified DM199 were either non-reduced (lanes 2, 3 & 4) or reduced (lanes 6, 7 & 8) with NuPAGE Sample Reducing Agent (Life Technologies, Carlsbad, CA) and loaded on to a NuPage Novex 4–12% BIS-TRIS pre-cast polyacrylamide gel (Life Technologies). The gel was stained with Colloidal Blue Staining (Life Technologies, Carlsbad, CA). Lanes 1, 5 and 9: molecular weight marker (SeeBlue Plus2 Standard, Life Technologies, Carlsbad CA).

DM199 was purified as a mixture of two glycoforms with a molecular weight range of 28–39 kDa (**Fig. 1**) and a theoretical pI of 4.6. The specific activity of DM199 was approximately 250 U/mg.

**Figure 2:**
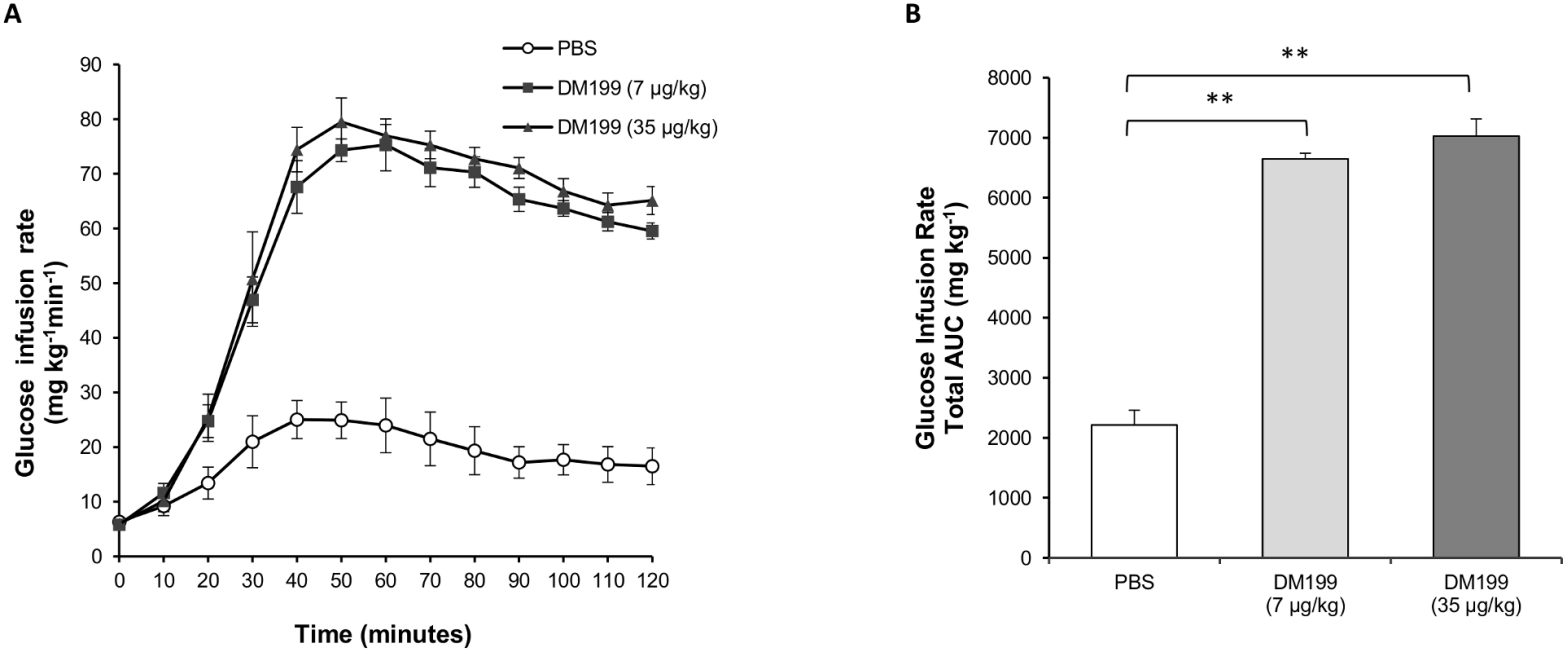
The effect of DM199 on glucose infusion rate during a hyperinsulinemic-euglycemic clamp. Catheterized Sprague-Dawley rats (n = 4; age 11–12 weeks) were injected s.c. with either PBS (Control), or DM199 (7 and 35 µg/kg) 30 minutes prior to commencement of a 120-minute hyperinsulinemic-euglycemic clamp (HEC). (A) Profile of glucose infusion rates during HEC. (B) Glucose infusion rate total AUC. Data are presented as the mean ± SEM, **p<0.01 vs. control.

The effect of DM199 on insulin sensitivity and glucose utilization was tested in a hyperinsulinemic euglycemic clamp (HEC) model in non-diabetic male Sprague-Dawley (SD) rats. Subcutaneous (s.c.) treatment with DM199 led to dramatically increased glucose infusion rates (GIR) compared to control although no significant difference between the two DM199 doses was detected (**Fig.** **2A** **)**. The AUC GIR was similarly significantly increased with the two doses of DM199 compared to control. There were no significant differences noted between the two doses of DM199 (**Fig. 2B**). Having demonstrated the efficacy of s.c. DM199 administration on glucose disposal in non-diabetic rats, the effects of DM199 on glucose disposal in animal models of T2D were evaluated.

### Acute Effects of DM199 in Diabetic *db/db* Mice

Diabetic female *db/db* mice were administered a single s.c. injection of test articles, and fasting blood glucose (FBG) levels were monitored over a 4-hour period. Insulin was used in this model as a positive control.

**Figure 3:**
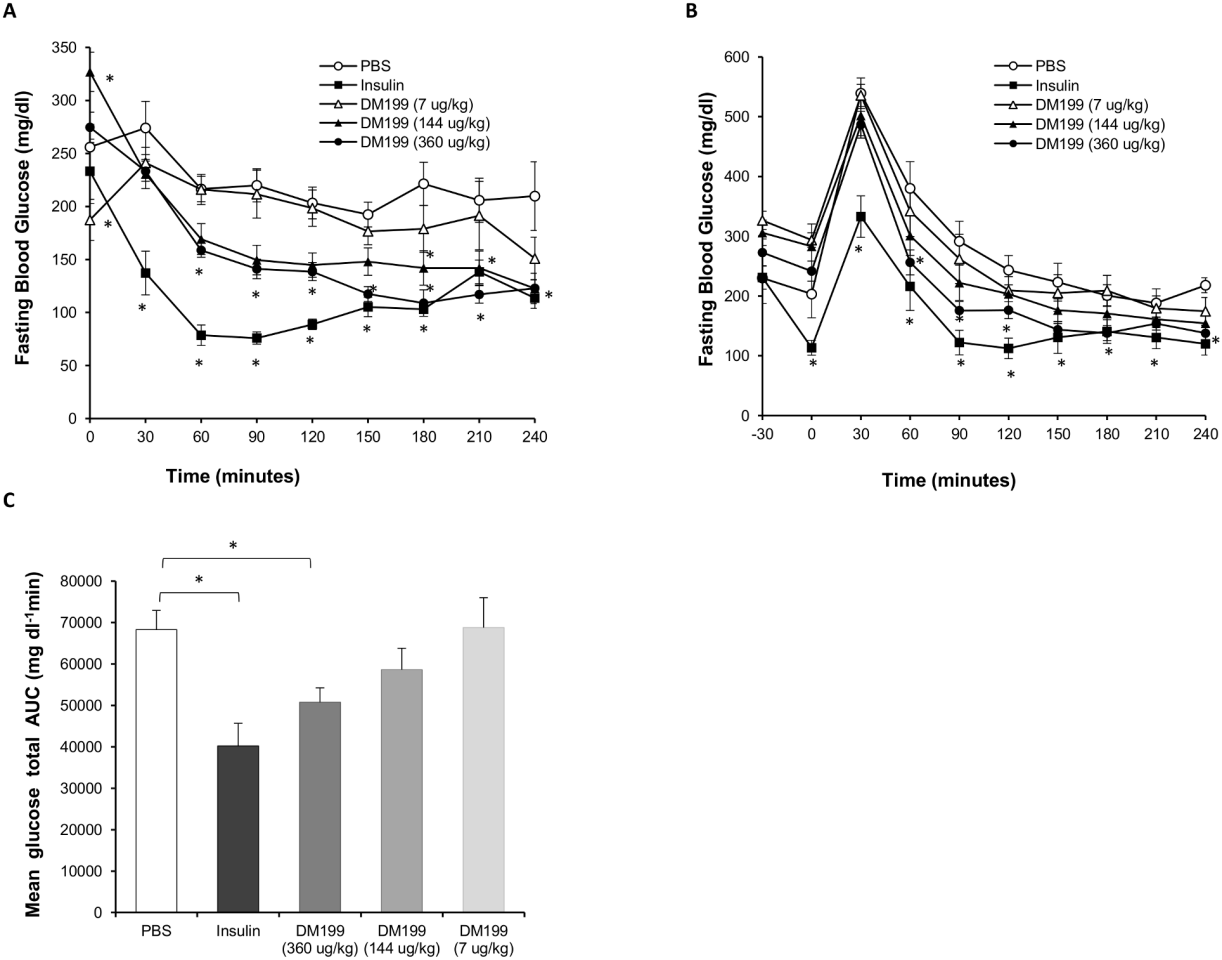
The effect of DM199 on fasting blood glucose and OGTT in *db/db* mice. (A) Blood glucose levels were measured in overnight-fasted female *db/db* mice (n = 9–10 per group) just prior to (t = 0 min) s.c. administration of PBS (Control), Insulin (5 U/kg) or DM199 (7, 144 or 360 µg/kg), and subsequently every 30 min over a 240 minute period. (B) After 7 days, the same cohorts of mice were fasted overnight, injected with test substances as in (A), and after 30 minutes, orally gavaged with 2 g/kg of glucose solution. Blood glucose was measured just prior to s.c. administration and oral gavage, and subsequently at 30 min intervals over a 240 minute period. (C) Mean glucose total AUC was computed from data in (B). Data are presented as the mean ± SEM. *p<0.05 vs. control.

At the start of the experiment we observed that the baseline FBG levels of the DM199 low (7 µg/kg) and medium (144 µg/kg) dose groups were significantly different from the vehicle-treated group (*p<*0.05; Fig. 3A). These significant differences in baseline values confounded further comparisons involving the low and medium DM199 dose groups. Insulin at 5 U/kg rapidly reduced FBG to normoglycemic levels within 60 minutes (**Fig. 3A**). The DM199 high dose group (360 µg/kg) significantly lowered FBG compared to PBS controls (*p<*0.05) within 60 minutes, although the decline was not as rapid as with insulin.

OGTTs were performed on the same cohorts of animals 6 days following the FBG tests (**Fig. 3B**). There were no differences in FBG between groups just prior to test article administration (t = −30). Following test article administration (t = 0), a significant drop in FBG was noted only for the insulin treated group. Relative to the PBS control group, only the insulin-treated group showed a significant decrease in the postprandial blood glucose peak at 30 minutes (*p<*0.001). Total glucose area under the curve (AUC) for animals treated with insulin and DM199 at the high dose were significantly lower than PBS control mice (**Fig. 3C**). The anti-diabetic effect noted in *db/db* mice was consistent with the increase in glucose disposal observed in the HEC experiment in SD rats.

### Effect of DM199 on Diabetic male ZDF *fa/fa* Rats

#### Acute effect of DM199 treatment

**Figure 4:**
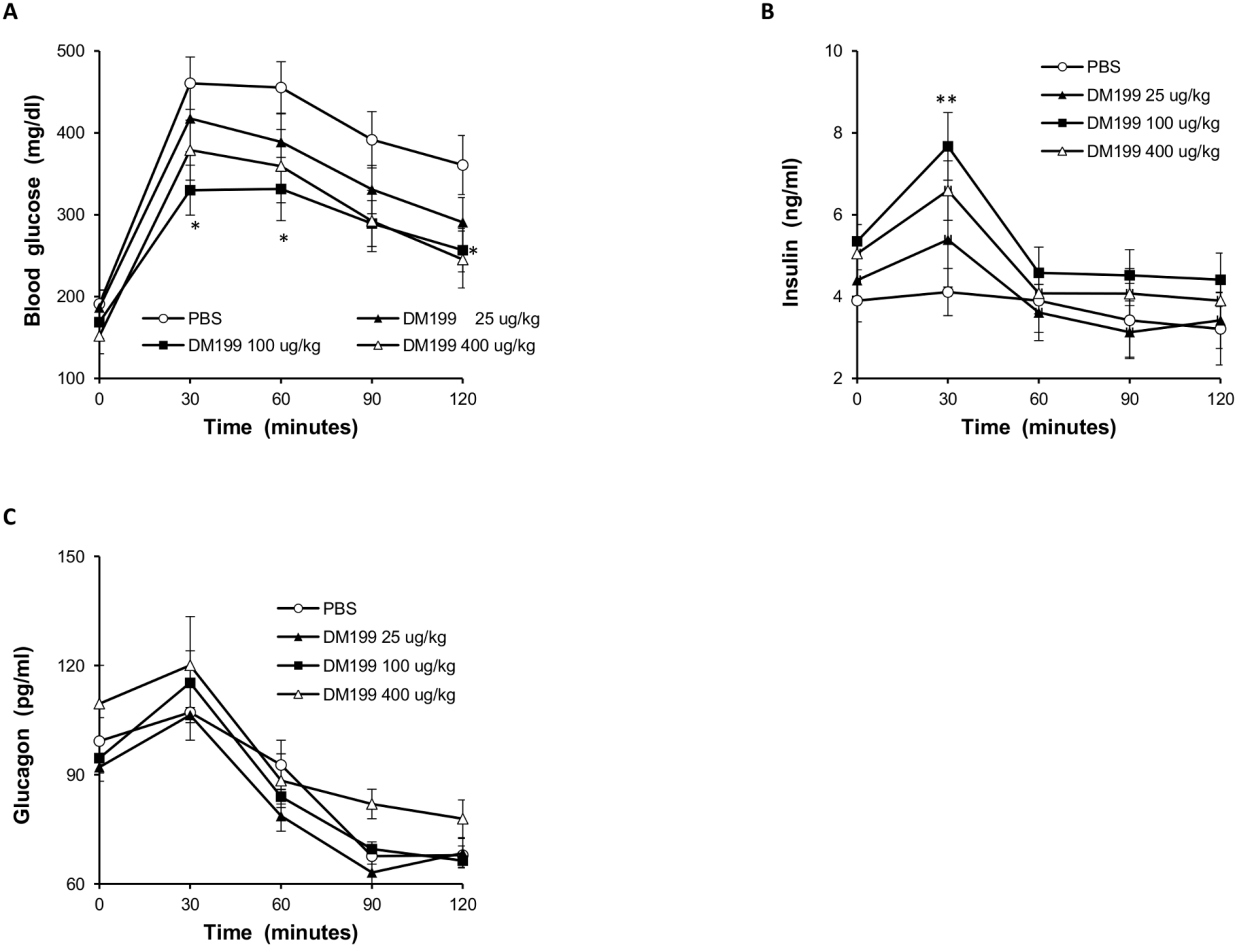
Effects of acute DM199 dosing in ZDF rats. 11-week old male ZDF rats (n = 8) were fasted for 12 hrs, fasting blood glucose was measured, and rats were injected s.c. with either PBS (control) or DM199 (25, 100 or 400 µg/kg). After 120 min rats were gavaged with 2 g/kg glucose solution (t = 0), and blood samples were drawn at 30-min intervals over a 120-minute period. (A) Average blood glucose levels before and during OGTT. (B) Average plasma insulin levels during OGTT. (C) Average plasma glucagon levels during OGTT. Data are presented as the mean ± SEM. *p<0.05; **p<0.01 vs. control.

**Figure 5:**
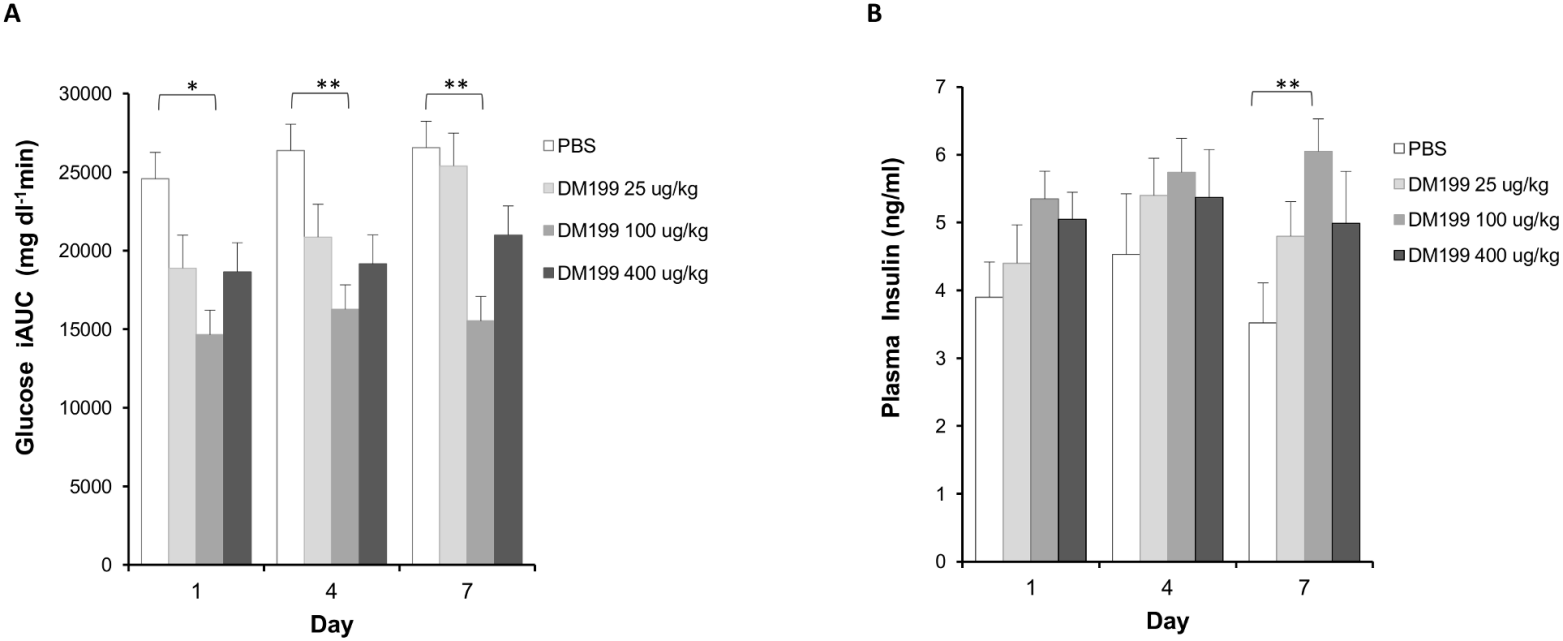
Effects of sub-acute DM199 dosing in ZDF rats. At 72-hour intervals (Day 1, 4 and 7), 11-week old male ZDF rats (n = 8) were fasted overnight (12 hrs.) and injected s.c. with either PBS (Control) or DM199 (25, 100 or 400 µg/kg). (A) After 120 min, rats were gavaged with 2 g/kg glucose solution, blood was sampled just prior to oral gavage (t = 0), and subsequently at 30 min intervals over a 120 minute period. For each dosing, glucose iAUC was calculated using t = 0 glucose value as background. (B) Average plasma insulin levels from fasted rats (t = 0). *p<0.05; **p<0.01 vs. control.

To further explore the dose-dependent anti-hyperglycemic responses to DM199, we measured FBG levels and conducted OGTTs to measure blood glucose, insulin and glucagon levels in ZDF *fa/fa* rats. Since FBG levels in DM199 treated mice were observed to trough only 120 minutes post-administration, all OGTTs in rats commenced two hours post-administration of DM199. Just prior to the start of the OGTT (t = 0 min), no significant differences in FBG, insulin or glucagon levels were evident with any of the DM199 doses (**Fig. 4** **A, B, C**). At 30 min. post-gavage, all three DM199 doses showed a decrease in peak glucose levels compared to control (**Fig. 4A**). Rats treated with 100 µg/kg DM199 also showed significantly reduced glucose levels at 60 and 120 min. post-gavage, and presented a significantly reduced glucose AUC (**Fig. 5A**) compared to vehicle-treated rats (∼40%, *p<*0.05). Insulin levels with all three doses of DM199 peaked at 30 minutes (**Fig. 4B**). The differences in peak glucose and insulin in both instances were significant only at the 100 µg/kg dose compared to control (*p<*0.05 and *p<*0.01 respectively). No effects on insulin AUC were noted (data not shown). No significant differences in glucagon levels were observed at any time point between the DM199 treatment arms and the control (**Fig. 4C**).

#### Sub-acute effect of DM199 treatment

**Figure 6:**
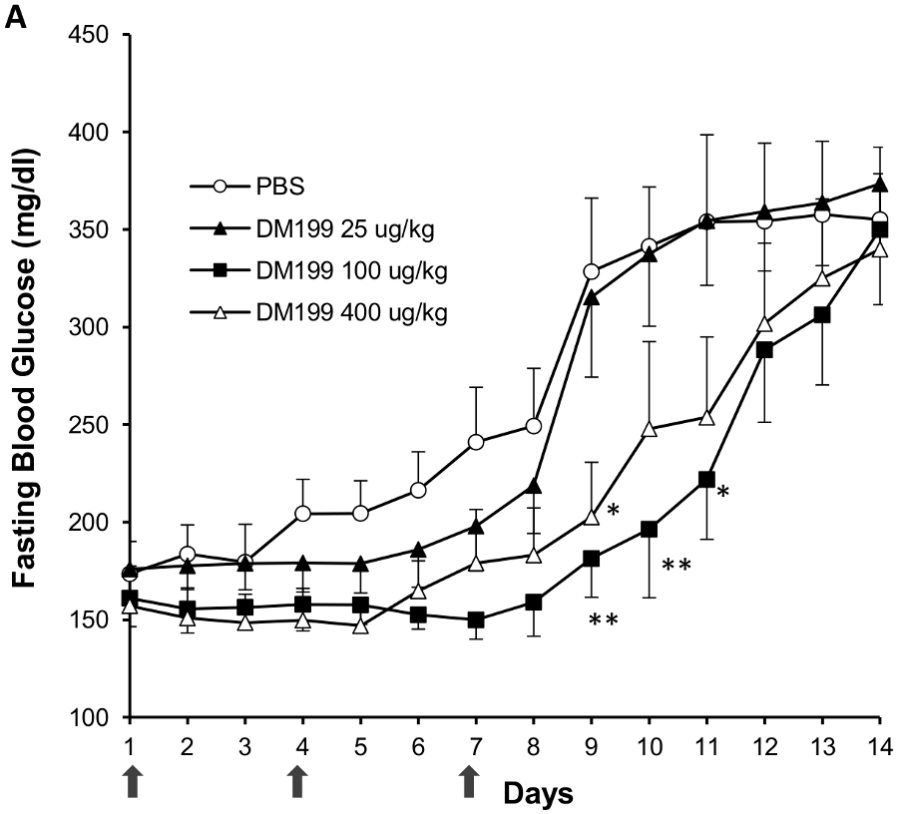
Effects of sub-acute DM199 dosing and drug wash-out. 11-week old male ZDF rats (n = 8) were fasted overnight (12 hrs.) and injected s.c. with either PBS (Control) or DM199 (25, 100 or 400 µg/kg) on days 1, 4 and 7 (arrows), with no subsequent treatments for a 7 day period. Fasting blood glucose levels were measured daily just prior to treatment. *p<0.05; **p<0.01 with respect to control treatment.

To test the effect of repeated DM199 administration on glucose and insulin levels, the study was extended in the same cohort of rats by administering DM199 twice more at 72-hour intervals (Day 4 and Day 7). OGTTs were conducted on treatment days, two hours post-dosing. Statistically significant reductions (*p*<0.05) in the OGTT incremental glucose AUC (∼40%) were observed on both Days 4 and 7 with the 100 µg/kg dose of DM199 (**Fig. 5A**). During the week-long treatment period, FBG levels gradually increased in the PBS control group, whereas the FBG levels stabilized in the high (400 µg/kg) and medium (100 µg/kg) DM199 dose groups, although no statistically significant differences were observed (**Fig. 6**). Animals were hyperinsulinemic and highly insulin resistant at the start of the experiment (typical HOMA1-IR >40) and there were no treatment related changes in the HOMA1-IR over the seven-day treatment period. There was a statistically significant increase in the fasting insulin levels between the 100 µg/kg dose of DM199 and PBS on Day 7 (*p<*0.05; **Fig. 5B**). At Day 7, the increased circulating insulin concentrations and lower FBG levels in DM199 treated rats compared to controls, translated into a statistically significant increase in the HOMA1-%B score for the 100 µg/kg DM199 dose group compared to the control (831.5+/−188.1 vs. 232.9+/−45.1 respectively; *p<*0.01).

#### Persisting effect of DM199 following cessation of treatment

To assess the effects of DM199 during a washout period, treatment with DM199 was discontinued after the third dose (Day 7). Fasting blood glucose levels were monitored daily for another 7 days. FBG levels on Day 8 in DM199 treated rats continued to remain lower than levels in PBS treated rats, and on Day 9, the differences were significantly lower in the 100 and 400 µg/kg doses compared to PBS (*p<*0.05). On Days 10 and 11, only the 100 µg/kg dose group showed significantly lower FBG levels (**Fig.** **6**). Thereafter FBG levels steadily rose to control levels by Day 14. These results support a persisting effect of DM199 in maintaining FBG for up to three days following dosing.

## Discussion

Diabetes remains a major global health problem despite several recently approved treatments. There remains a large unmet need for new therapeutics with unique mechanisms of action to treat this multi-faceted disease. Modulation of the kallikrein-kinin system (KKS) has been proposed as a novel approach to treating T2D and associated complications [17], [18]. Several *in* *vitro* and *in* *vivo* studies suggest that BK-mediated mechanisms may contribute to improved insulin sensitization and glucose homeostasis. Treatment with KLK-1, and stimulation of the BKR2 with BK, leads to positive effects on insulin release, sensitivity and glucose uptake in cultured adipocytes, skeletal muscle cells, and freshly isolated tissues [19]–[21]. BK potentiates the effects of insulin by enhancing the phosphorylation of key intermediates in the PI3 kinase-Akt signaling pathway and the translocation of GLUT4 glucose transporters to the cell membrane [6], [7], [22], [23]. In concordance with the observed *in* *vitro* effects, chronic administration of BK to Zucker diabetic rats significantly improved whole body glucose tolerance and insulin sensitivity [24]. BK is a potent effector of nitric oxide (NO) bioavailability in endothelial cells. Activation of PI3 kinase-Akt and PLC-β signaling cascades downstream of the BKR2 stimulates NO synthesis and prostacyclin release, resulting in vasodilation [25], [26]. In addition to the beneficial effects of NO-mediated vasodilation on blood pressure homeostasis, several studies (reviewed in [27]) suggest that optimal stimulation of PI3 kinase-Akt-NO pathway is critical for the various protective effects of insulin signaling that are impaired or completely inhibited in diabetes.

Support for the role of the KKS as a therapeutic target in T2D and associated complications can be inferred from the clinical experience with angiotensin converting enzyme (ACE) inhibitors which improve insulin sensitivity and glucose control in T2D patients [28]. ACE inhibitors exert their pharmacological effects in part through the KKS, by inhibiting kinin degradation, resulting in higher kinin levels [29]. Since KLK-1 is an integral part of the KKS, we hypothesized that administration of human KLK-1, which generates kinins *in* *situ*, could have anti-diabetic effects. Previous reports indicate that human KLK-1 generates kinins from kininogens in mice and rats [30], [31] and that delivery of the gene for human KLK-1 into these rodents has beneficial effects on glucose control and insulin resistance [11], [13]. Herein we report for the first time, studies demonstrating that administration of a purified recombinant form of human tissue kallikrein, DM199, possesses anti-hyperglycemic effects in multiple animal models of T2D.

Anti-diabetic agents typically reduce fasting blood glucose, post-prandial glucose or both as part of their anti-hyperglycemic effects. In *db/db* mice, acute administration of 360 µg/kg DM199 significantly reduced FBG levels suggesting a potent anti-hyperglycemic action, but did not significantly decrease the glucose peak during OGTTs. This may be due in part to the fact that mice were gavaged only 30 minutes after dosing of DM199 and that the maximal glucose-lowering effect of DM199, as seen in the FBG, is likely to occur only at or greater than 120 minutes post-administration. Both insulin and DM199 at the 360 µg/kg dose reduced the glucose total AUC suggesting that DM199 has a demonstrable effect on post-prandial glucose control. Having observed an acute anti-diabetic effect on FBG and post-prandial glucose levels in the *db/db* mouse, we then tested the anti-diabetic effects of sub-acute DM199 exposure in another model of T2D, the ZDF rat model.

In ZDF rats, the OGTTs were started two hours post-administration of the drug in contrast to 30 minutes post-administration in *db/db* mice. The two-hour time point was selected based on the time required for FBG to reach trough levels in the *db/db* mice. However, in contrast to the significantly reduced FBG levels in *db/db* mice, no significant reduction of FBG in ZDF rats was observed two hours after DM199 administration. The reason for the difference in the effect on FBG between the two models is not currently known.

In the hyperinsulinemic euglycemic clamp (HEC) study with DM199-treated non-diabetic rats, the dramatic increase in glucose infusion rates suggests that DM199 could be exerting an acute insulin sensitizing effect. This result is consistent with Yuan *et al.* who showed that virally delivered KLK-1 increased insulin sensitization in a streptozotocin-induced rat model of T2D [13]. Yuan *et al.* reported a low baseline HOMA-IR score (∼1.77) at the start of therapy and employed a considerably long (12 weeks) treatment period. Two lines of evidence from our experiments with ZDF rats suggest however, that the DM199-dependent improvement in glucose control is not due to increased insulin sensitivity. First, the ZDF rats were extremely insulin-resistant with a baseline HOMA1-IR greater than 40, and showed a negligible change in HOMA1-IR upon DM199 treatment. Second, acute administration and short (1 week) exposure to DM199, demonstrated strong positive effects on glucose control, as well as a gradual increase in fasting insulin levels. Differences in the diabetic models, therapeutic agents and/or treatment periods could additionally account for the disparity between our results and those of Yuan *et al*. The acute insulin sensitizing effect of DM199 observed in our HEC study, warrants further investigation.

At the end of a 7-day sub-acute dosing period (Day 7), ZDF rats treated with 100 µg/kg of DM199 showed significant increases in fasting insulin levels and HOMA1-%B scores compared to controls. FBG levels in this dose group remained significantly lower than controls on subsequent days (Days 9, 10 and 11) of a drug wash-out period. The week-long drug wash-out study (Days 8–14) was conducted when ZDF rats were 12 weeks old and are hyperinsulinemic, but their beta cell numbers are beginning to decrease [32]. We speculate that the escalating FBG levels in untreated rats on Days 8–14 is likely associated with a loss of beta cells and that the delayed rise in FBG levels of medium and high dose DM199-treated rats during the same period, may be due to a protective effect on beta cell function, or improved insulin secretion from the remaining viable beta cells. Higher fasting insulin levels and increased HOMA1-%B scores are consistent with both an insulin-secretion and a beta cell protective mechanism of action, although further studies are required for confirmation. Thiazolidinedione drugs and GLP-1 agonists have been shown to preserve beta cell mass and function in ZDF rats of equivalent age [33], [34]. Similar to GLP-1 agonists, the anti-hyperglycemic effects of DM199 are manifested through reduction in both FBG and post-prandial glucose levels. However in contrast to GLP-1 agonists [35], DM199 did not appear to reduce post-prandial glucagon levels.

Further studies are needed to better understand the mechanism of action of the anti-diabetic effects of DM199. It is likely that DM199 administration leads to the local generation of BK, subsequent engagement of the BKR2, and activation of signaling pathways that lead to enhanced glucose uptake in peripheral tissues [28]. However, the possibility that DM199 directly stimulates the BKR2 cannot be ruled out, as several investigators have reported the direct interaction of KLK-1 with BKR2. In cultured cells KLK-1 activated the BKR2, triggering Ca^2+^ and arachidonic acid release [36], even in the complete absence of kininogens [37]. In kininogen-deficient rats, KLK-1 exerted cardioprotective effects that were mediated by BKR2 and NO [38]. The direct interaction of KLK-1 with BKR2 was shown to have concentration-dependent effects on receptor activation and proteolytic degradation. Using HEK 293 cells transfected with rabbit BKR2, Houle *et al* showed that at low nanomolar concentrations, KLK-1 stimulated the BKR2, but at higher micromolar concentrations, receptor degradation and internalization occurred [39]. It is possible that the maximal effects observed with intermediate doses of DM199 to ZDF rats, are a reflection of the sensitivity of the rat BKR2 to DM199 proteolytic activity. The 100 µg/kg DM199 dose administered to ZDF rats was consistently the most effective in reducing blood glucose and in elevating insulin levels, compared to the higher 400 µg/kg dose (Figs. 4, 5 and 6). It remains to be seen if DM199 elicits *in* *vitro* concentration-dependent effects on the rat BKR2 similar to that observed by Houle *et al*. with KLK-1; these are the focus of ongoing studies. In summary, these studies suggest that DM199 exerts anti-hyperglycemic effects in animal models of T2D, and is a novel therapeutic candidate for T2D. Further elaboration of the mechanism of action of DM199 could include investigation of the effects of BKR2 antagonists such as HOE-140 [40] and bradyzide [41] on *in* *vivo* glucose disposal. Studies on the effect of DM199 on glucose uptake and utilization by skeletal muscles and other tissues would further characterize its mechanism of action. Additional toxicological and pharmacokinetic studies are warranted in order to further advance the protein towards clinical development.
